# Capturing Single Cell Genomes of Active Polysaccharide Degraders: An Unexpected Contribution of *Verrucomicrobia*

**DOI:** 10.1371/journal.pone.0035314

**Published:** 2012-04-20

**Authors:** Manuel Martinez-Garcia, David M. Brazel, Brandon K. Swan, Carol Arnosti, Patrick S. G. Chain, Krista G. Reitenga, Gary Xie, Nicole J. Poulton, Monica Lluesma Gomez, Dashiell E. D. Masland, Brian Thompson, Wendy K. Bellows, Kai Ziervogel, Chien-Chi Lo, Sanaa Ahmed, Cheryl D. Gleasner, Chris J. Detter, Ramunas Stepanauskas

**Affiliations:** 1 Bigelow Laboratory for Ocean Sciences, West Boothbay Harbor, Main, United States of America; 2 Colby College, Waterville, Main, United States of America; 3 Department of Marine Sciences, University of North Carolina, Chapel Hill, North Carolina, United States of America; 4 Genome Science Group, Los Alamos National Laboratory, Los Alamos, New Mexico, United States of America; 5 Microbial and Metagenome Program, Joint Genome Institute, Walnut Creek, California, United States of Aemerica; Institute for Genome Sciences, University of Maryland School of Medicine, United States of America

## Abstract

Microbial hydrolysis of polysaccharides is critical to ecosystem functioning and is of great interest in diverse biotechnological applications, such as biofuel production and bioremediation. Here we demonstrate the use of a new, efficient approach to recover genomes of active polysaccharide degraders from natural, complex microbial assemblages, using a combination of fluorescently labeled substrates, fluorescence-activated cell sorting, and single cell genomics. We employed this approach to analyze freshwater and coastal bacterioplankton for degraders of laminarin and xylan, two of the most abundant storage and structural polysaccharides in nature. Our results suggest that a few phylotypes of *Verrucomicrobia* make a considerable contribution to polysaccharide degradation, although they constituted only a minor fraction of the total microbial community. Genomic sequencing of five cells, representing the most predominant, polysaccharide-active *Verrucomicrobia* phylotype, revealed significant enrichment in genes encoding a wide spectrum of glycoside hydrolases, sulfatases, peptidases, carbohydrate lyases and esterases, confirming that these organisms were well equipped for the hydrolysis of diverse polysaccharides. Remarkably, this enrichment was on average higher than in the sequenced representatives of *Bacteroidetes*, which are frequently regarded as highly efficient biopolymer degraders. These findings shed light on the ecological roles of uncultured *Verrucomicrobia* and suggest specific taxa as promising bioprospecting targets. The employed method offers a powerful tool to rapidly identify and recover discrete genomes of active players in polysaccharide degradation, without the need for cultivation.

## Introduction

Polysaccharides are major components of biomass and detritus in aquatic ecosystems and their microbial degradation constitutes one of the key bottlenecks in the carbon cycle [1], [2]. Better understanding of the microbial types and their biochemical machinery involved in the degradation of polysaccharides is also of special interest for cost-effective biofuel production from terrestrial plants and algae [3]–[5]. Laboratory-based experiments on cultured isolates have been traditional sources of information on polysaccharide-degrading microbial taxa and enzymes, but they represent only a minor fraction of the active players in the carbon cycling in nature [6]. Culture-independent methods, such as microautoradiography coupled to fluorescent *in situ* hybridization, have provided valuable insights into the uptake rates of some organic compounds by broad microbial phylogenetic groups [7]. More recently, deep metagenomic sequencing has been proven effective in high-throughput discovery of individual polysaccharide hydrolysis genes [8]–[10]. However, methodological limitations have so far hindered unambiguous identification of microbial taxa responsible for specific hydrolytic processes in the environment and the recovery of entire carbohydrate degradation pathways from members of the microbial “uncultured majority".

To address this challenge, we developed a novel research approach, which relies on fluorescent labeling of polysaccharides of interest [11] and the use of these polysaccharides in samples taken directly from the environment to label uncultured microbial cells involved in polysaccharide hydrolysis. Subsequent single-cell genomic DNA amplification and sequencing then yields detailed insight into the metabolic potential of the labeled microorganisms. We employed this approach to analyze freshwater and coastal bacterioplankton for degraders of laminarin and xylan, two of the most abundant storage and structural polysaccharides in nature [12]. Bacterial breakdown of these polysaccharides has been widely demonstrated in aquatic environments [2], but the identity of specific microbes performing this process *in situ* has remained largely unknown [13], due to the challenges outlined above.

## Results and Discussion

### Overall strategy

Cells that probe positive for a specific polysaccharide are detected and separated from the rest of the natural microbial assemblage by fluorescence-activated cell sorting. Individual, polysaccharide-positive cells are deposited into microplates and subjected to high-throughput single-cell genomic DNA amplification and sequencing [14]–[18]. We refer to this technique as Fluorescent Substrate Single Amplified Genome Analysis (FS-SAGA).

### Optimization of conditions for cell probing with fluorescent polysaccharides

**Figure 1:**
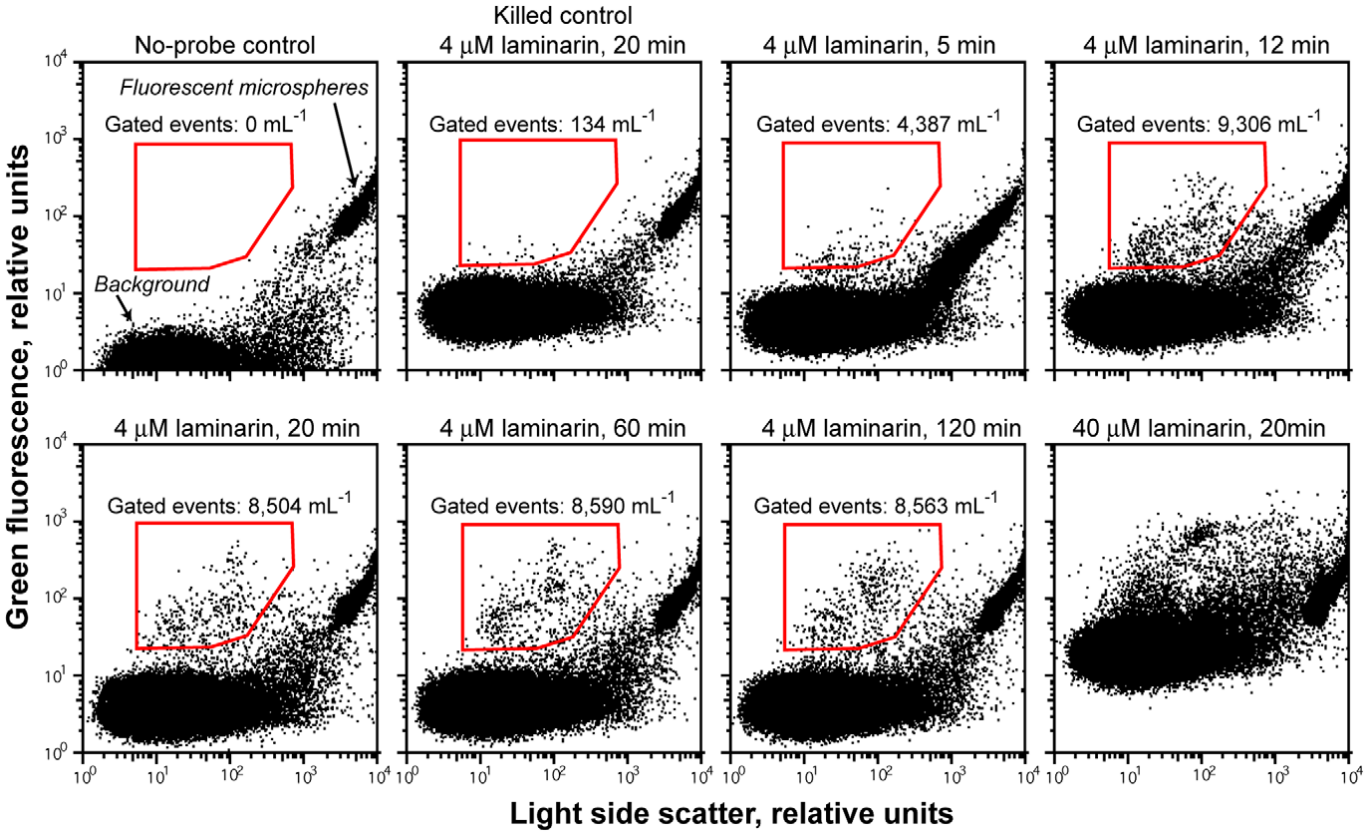
Optimization of cell probing conditions with fluorescently labeled laminarin. Flow cytometric dot plots of heat-killed and live freshwater samples incubated for various lengths of time with 4 or 40 µM fluorescently labeled laminarin. Red polygons indicate gates used to count putative laminarin-positive cells.

Bacteria-size particles with green fluorescence were detected in aquatic samples that were amended with either 4 or 40 µM fluoresceinamine-labeled laminarin (Figure 1). The number of putative fluorescent cells in 4 µM laminarin treatments increased between 5 and 12 minutes and was stable for the remaining two hours of incubation. The heat-killed control had over 60-fold lower abundance of particles with elevated fluorescence in the gated area compared to the live treatments. No fluorescent particles were detected in the gated area in the live control treatment without the addition of the fluoresceinamine-labeled laminarin. Compared to the 4 µM laminarin treatments, 40 µM treatments had significantly higher background fluorescence, obscuring the demarcation of labeled microbial cells. Thus, a 12–120 minute incubation with 4 µM fluoresceinamine-labeled laminarin was optimal for bacterioplankton probing.

### Phylogenetic composition of single amplified genomes

**Figure 2:**
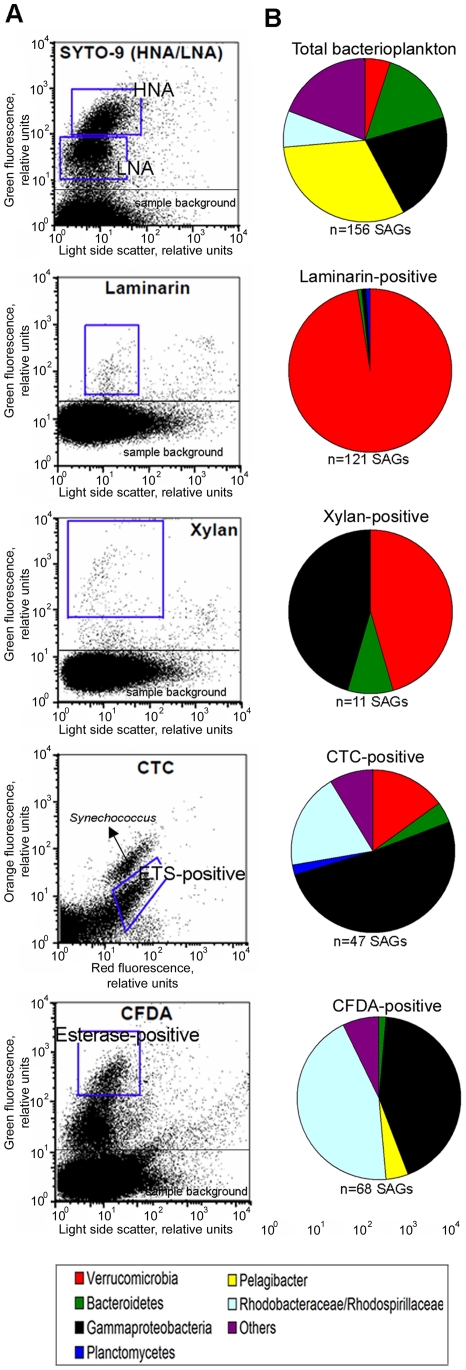
Flow-cytometric sort gates (A) and taxonomic composition (B) of single amplified genomes (SAGs) generated from coastal bacterioplankton using various fluorescent probes. Bacterioplankton were probed with (from top to bottom): 1) nucleic acid stain SYTO-9, targeting high- and low-nucleic acid content cells (HNA and LNA cells) representing a random subset of the entire microbial assemblage; 2) fluorescently-labeled laminarin; 3) fluorescently-labeled xylan; 4) 5-cyano-2,3-ditolyltetrazolium chloride (ETS-active cells) and 5) carboxyfluoresceindiacetate (esterase-active cells). Gates used for cell sorting are indicated in blue.

Using a combination of single cell fluorescence-activated cell sorting, whole genome multiple displacement amplification and subsequent PCR and sequencing of the 16S rRNA genes, we generated and identified 414 coastal and 68 freshwater single amplified genomes (SAGs; Figures 2 and S1, Table S1). In both environments, the composition of SAGs generated from cells labeled with the generic DNA stain SYTO-9 was consistent with prior findings of total bacterioplankton composition using other culture-independent techniques. The SAR11 cluster, *Bacteroidetes*, and *Gammaproteobacteria* dominated coastal SAGs (Figure 2B) [19], while *Betaproteobacteria Polynucleobacter* spp., *Actinobacteria* acI, *Alphaproteobacteria* LD12 cluster and Bacteroidetes dominated freshwater SAGs (Figure S1) [20]. In contrast, SAGs generated from laminarin-labeled cells were dominated by *Verrucomicrobia* in both coastal and freshwater samples (Figure 2B and Figure S1). Other laminarin-positive cells belonged mostly to the *Bacteroidetes, Planctomycetes, and Gammaproteobacteria* (Figure 2 and S1, S2). Only 11 coastal and 5 freshwater, xylan-positive SAGs produced SSU rRNA gene sequences. The xylan-positive SAGs were dominated by *Verrucomicrobia* and *Gammaproteobacteria* (including the SAR86 cluster) in the coastal sample and by *Verrucomicrobia* in the freshwater sample.

**Figure 3:**
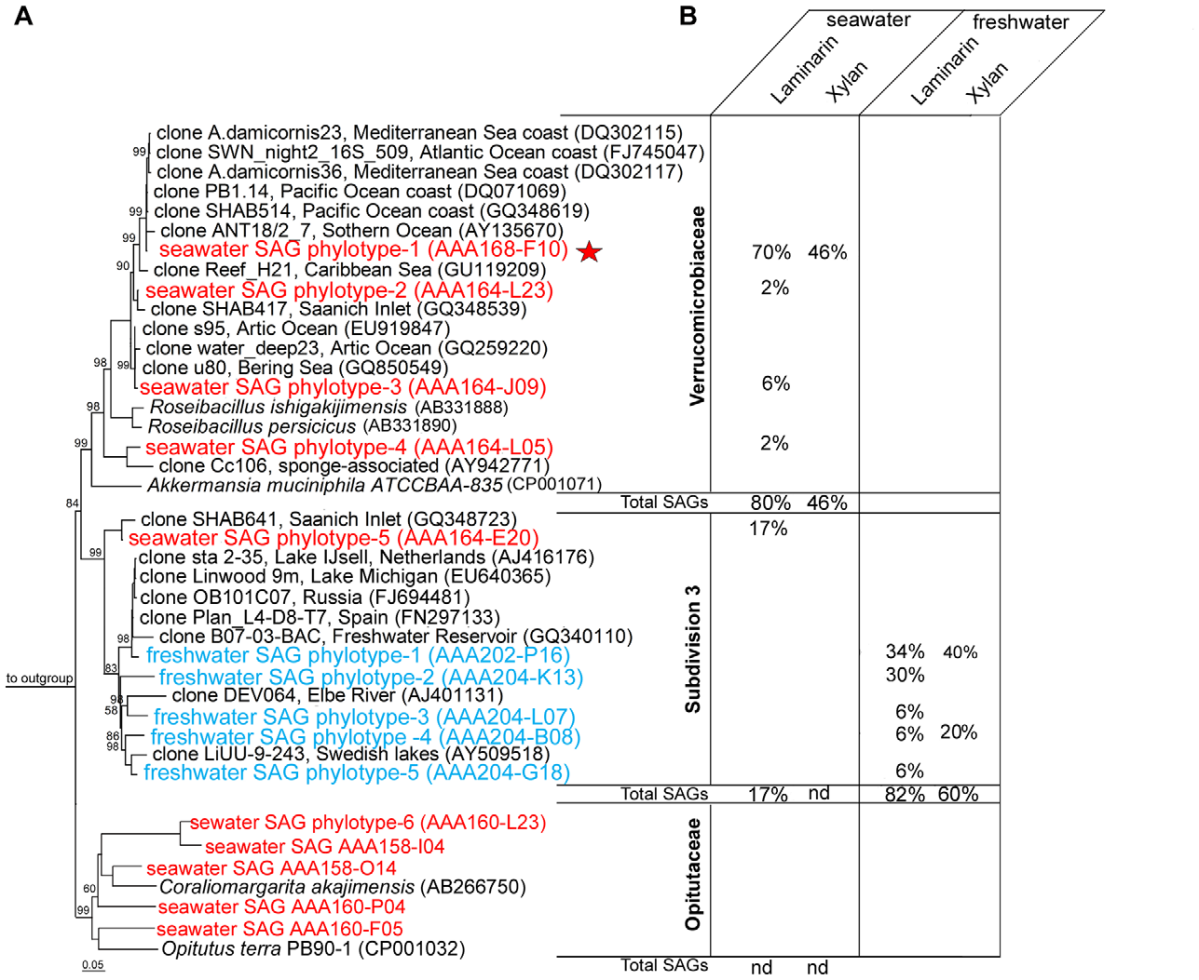
Phylogenetic composition of *Verrucomicrobia* SAGs. (A) Maximum likelihood phylogenetic analysis of the SSU rRNA gene sequences. Bootstrap (1000 replicates) values ≥50 are displayed. Each phylotype, indicated in red (coastal) or blue (freshwater) is formed by SAGs with ≥99% SSU rRNA gene sequence similarity. Five SAGs from the most abundant putative polysaccharide degrader phylotype in the coastal sample were selected for whole genome sequencing (red star). (B) Phylotype relative abundances in SAG libraries generated using various fluorescent probes. (nd) = not detected in a SAG library.

There was a clear phylogenetic separation between coastal and freshwater *Verrucomicrobia* SAGs, with *Verrucomicrobiacea* dominating the coastal sample and Subdivision 3 dominating the freshwater sample (Figure 3). *Verrucomicrobia* SAGs grouped into ten marine and five freshwater phylotypes sharing ≥99% SSU rRNA gene identity within each phylotype. Of them, one marine phylotype (AAA168-F10) and two freshwater phylotypes (AAA202-P16 and AAA204-K13) comprised over 2/3 of polysaccharide-positive SAGs in their respective environments. Sequences that were identical or closely related to the most abundant marine and freshwater phylotypes (AAA168-F10 and AAA202-P16) have been reported from other environments, indicating that they are broadly distributed and are not limited to the samples analyzed in this study (Figure 3A). Remarkably, none of these phylotypes comprised more than 1% of the total bacterioplankton (HNA and LNA fractions). This corroborates our finding that only ∼0.1% of bacterioplankton cells retained laminarin and xylan fluorescence in both aquatic environments. Our results suggest unexpected roles of uncultured *Verrucomicrobia* phylotypes as active laminarin and xylan degraders in the coastal and freshwater environments examined in this study.

To determine the composition of metabolically active members of the studied coastal microbial assemblage, we labeled them with the electron transport system (ETS) activity probe 5-cyano-2,3-ditolyltetrazolium chloride (CTC) and the esterase activity probe carboxyfluorescein diacetate (CFDA), both of which are often used in microbial ecology studies [21], [22]. SAGs were generated from the labeled cells and identified by their SSU rRNA gene sequencing. The composition of SAGs generated from esterase- and ETS-positive coastal bacterioplankton was similar to each other and was enriched in *Gamma-* and *Alphaproteobacteria* (*Rhodospirillaceae* and *Rhodobacteraceae*) relative to the total bacterioplankton (Figure 2B, S3). In the costal sample, the most abundant polysaccharide-positive *Verrucomicrobia* phylotype AAA168-F10 constituted 6% of ETS-positive SAGs (Table S2), providing further evidence that this phylotype was a metabolically active member of the microbial assemblage. Due to the potential toxicity of CTC to some microbial cells, its relevance in microbial ecology studies has been actively debated [21]–[24]. The compositional similarity between ETS-positive and esterase-positive SAGs observed in this study suggests that both probes detect the same taxonomic groups, likely representing the most metabolically active members of the microbial community.

### Whole genome analysis

**Figure 4:**
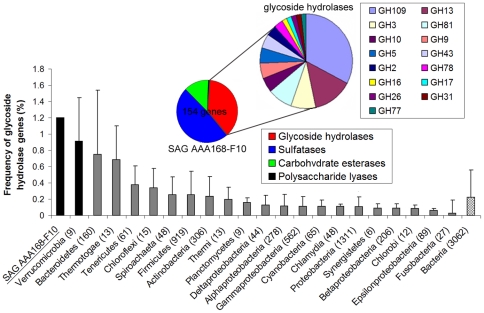
Comparative analysis of genes encoding hydrolytic enzymes in prokaryote genomes. The bar chart indicates the genome-wide frequency of glycoside hydrolase genes in various microbial groups, average ± standard deviation. The number of publicly available genomes found in the IMG database (as of February 2012) for each taxonomic group is provided in parentheses. The average enrichment of glycoside hydrolases was also estimated for the *Bacteria* domain. The small pie chart shows the number and composition of genes involved in polysaccharide hydrolysis in the *Verrucomicrobia* SAG AAA168-F10. The large pie chart shows CAZy families of glycoside hydrolase genes detected in SAG AAA168-F10. Each glycoside hydrolase family is indicated as GH-xxx, according to CAZy database nomenclature [25].

To verify the potential role of the most abundant polysaccharide-positive *Verrucomicrobia* phylotype AAA168-F10, we performed genomic sequencing of five SAGs representing this phylotype, employing a combination of GAIIx (Illumina) and PacBio™ RS (Pacific Biosciences) sequencing technologies. The obtained assemblies ranged 1.0–4.9 Mbp, with estimated 32%–88% genome recovery (Table S3). The fraction of genome encoding various carbohydrate-active enzymes was almost identical in all five SAGs, and the number of glycoside hydrolase genes correlated with the genome size (R^2^ = 0.93; Figure S4), indicating that the number of glycoside hydrolases was a function of genome coverage in the five sequenced SAGs. The five SAGs shared high degree of average nucleotide identity (ANI; >97.8%) and similar tetranucleotide signature frequencies (>0.96; Figure S5), further confirming that the SAGs were closely related. Therefore, we focused our further annotation efforts on SAG AAA168-F10, which had the largest fraction of the genome recovered. First, we searched for genes encoding glycoside hydrolases, which catalyze the initial step of converting high molecular weight polysaccharides into oligo- or monosaccharides that are sufficiently small (<600 Da) to be transported into the cell for further processing [25]. We found that *Verrucomicrobia* in general and AAA168-F10 in particular were enriched in glycoside hydrolases (0.91% and 1.2% of total genes, respectively) when compared to the 3,062 publicly available bacterial genomes (Figure 4). On average, about 0.2% of bacterial genes encode glycoside hydrolases. Interestingly, the fraction of these genes in AAA168-F10 and other publicly available *Verrucomicrobia* genomes was on average higher than in *Bacteroidetes*, which are frequently regarded as the most efficient biopolymer degraders [7].

**Figure 5:**
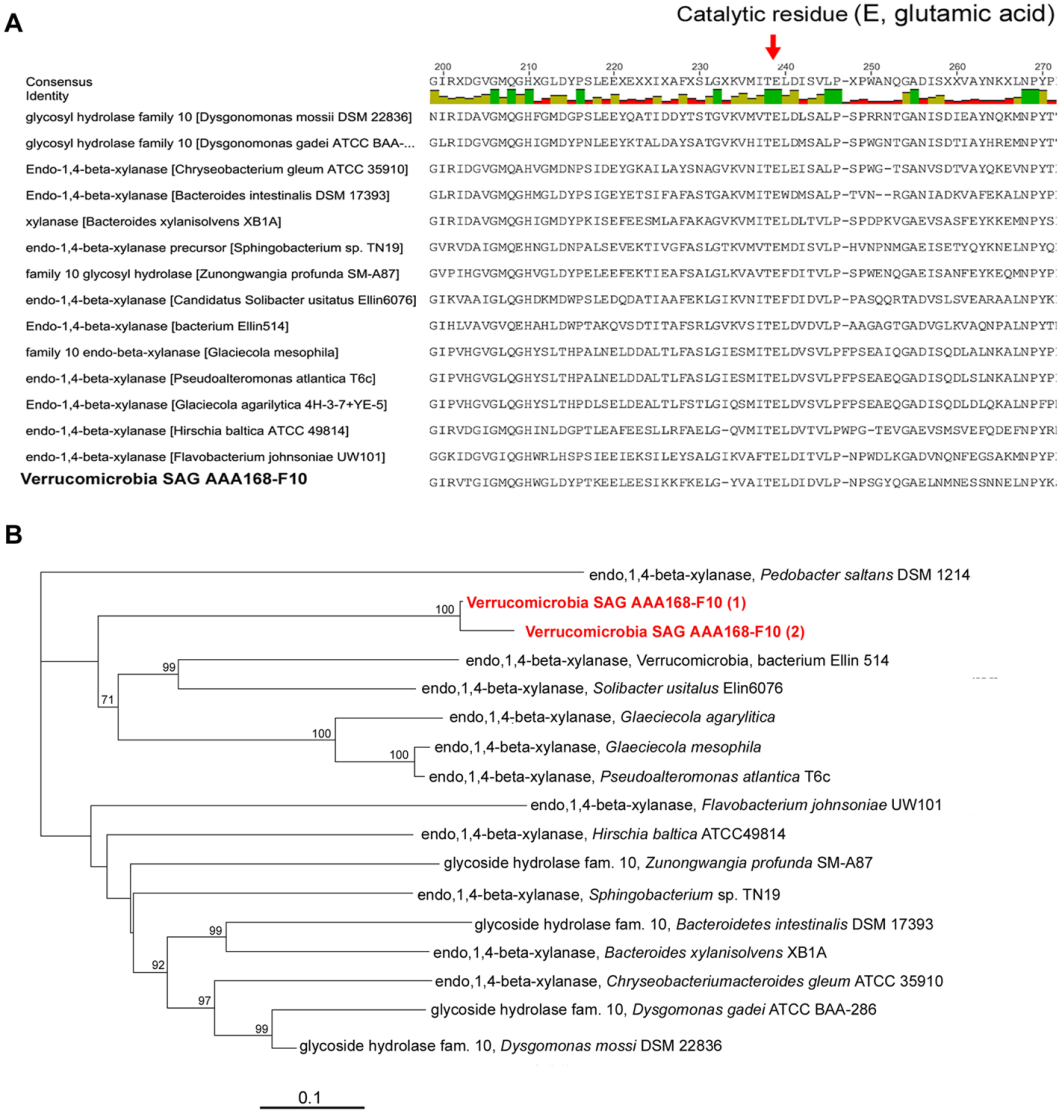
Evidence for the laminarinase gene in the single amplified genome AAA168-F10. (A) Active site, including the catalytic residues responsible for laminarin hydrolysis, derived from Conserved Domain Protein, SWISS-MODEL, and PROSITE databases. (B) Neighbor-joining phylogenetic tree of amino acid sequences, applying the Kimura evolutionary model and indicating bootstrap values above 50.

Genome sequence analysis confirmed that AAA168-F10 possesses the genes encoding both laminarinase and xylanase, including their active sites and catalytic residues (Figures 5 and S6). We also detected signal peptide cleavage sites at the N-termini of these proteins, which direct the protein's outward transport across the cellular membrane (Figure S7). The SAG AAA168-F10 genome contained 58 putative glycoside hydrolases representing 15 carbohydrate-active enzyme (CAZy) families [26] (Figure 4). These enzymes are potentially involved in the degradation of complex and diverse biopolymers, including mucopolysaccharides, glycoproteins, peptidoglycan, celluloses, hemicelluloses, and glycogen (Table S4). Furthermore, the AAA168-F10 genome encoded an exceptional number of sulfatases (75 genes; Figure 4). These enzymes have been proposed to be involved in the hydrolysis of sulfate groups to access the carbon skeleton of sulfated polysaccharides, which are major constituents of algal cell walls [27]. In addition, AAA168-F10 contained a significant number of carbohydrate lyases and esterases (Figure 4), which complement the enzymatic activity of glycoside hydrolases to degrade polysaccharides [26]. We also detected 199 peptidase genes, representing 67 protein families, indicating a vast proteolytic potential (Figure S8). Among detected peptidases are members of the M23B family, which is likely involved in the lysis of bacterial cell wall peptidoglycans [27]. The detected M23B peptidases contained signal peptide cleavage sites indicative of periplasmic or extracellular secretion [28]. Thus, genome sequence analysis provides strong support for the hypothesis that *Verrucomicrobia* phylotypes captured using the FS-SAGA are well equipped for the hydrolysis of diverse polysaccharides and other complex biopolymers.

Prior studies employing cultivation, genomics, metagenomics and radiolabeled DOM uptake assays have suggested the importance of *Bacteroidetes, Clostridia, Planctomycetes, Spirochaetes*, and *Gammaproteobacteria* in polysaccharide degradation in aquatic, soil and cow-rumen environments [7], [27], [29]. In contrast, very little is known about the metabolism and ecological roles of *Verrucomicrobia*, primarily due to the difficulty in isolation and subsequent paucity of experimental and genomic data. Members of this phylum are widespread in aquatic, terrestrial and intestinal tract environments [30]–[33] and have been found in association with algae, protozoa, and invertebrate animals [34]–[36]. To the best of our knowledge, only one prior report shows the ability of *Verrucomicrobia* to degrade polysaccharides, employing cultures isolated from soils [37]. Our study suggests that previously unrecognized, uncultured and relatively rare taxa of *Verrucomicrobia* are likely highly active polysaccharide degraders in the studied marine and freshwater environments.

### Methodological considerations

The significant enrichment of the sequenced SAGs in genes involved in polysaccharide degradation provides support for FS-SAGA as a useful tool to recover genomes of active polysaccharide degraders, without the need for cultivation. The striking difference in the taxonomic composition of polysaccharide-positive SAGs and esterase- and ETS-positive SAGs provides further evidence that cell labeling by fluorescent polysaccharides targeted microbial groups that express very specific physiological traits rather than general viability. We can rule out the possibility of applied probes labeling *Verrucomicrobia* due to their cell wall peculiarities rather than enzymatic activity, because: a) closely related *Verrucomicrobia* phylotypes, expected to have similar cell wall structure, exhibited highly divergent responses to the same substrate (Figure 3) and b) no cells exhibited fluorescence in killed control treatments (Figure 1).

The FS-SAGA approach offers significant advantages compared to other culture-independent techniques for the discovery and genomic analysis of biopolymer degraders. Compared to metagenomic sequencing, advantages include a) targeting members of natural microbial assemblages that are highly active in the degradation of specific polymers under the studied conditions (*in situ* or manipulated), b) recovery of near-complete genomes, independent of the complexity of the microbial community and the relative abundance of the target taxa, c) a physiology- rather than genetics-based cell targeting, making it independent of existing, limited genetic databases, and d) fast cell probing, removing the risk of biasing microbial composition and confusing primary and secondary responses to the substrate amendment.

The FS-SAGA is not exempt from limitations. We assume that laminarin- and xylan-positive cells retain the fluorescently-labeled polysaccharides on their cell surface, presumably through enzyme-associated carbohydrate binding domains [38]. Some active biopolymer degraders may thus fail to retain the fluorescently- labeled polysaccharide if they lack distinct carbohydrate binding domains, or their enzymes are released into the surrounding medium rather than attached to the cell surface or contained in the periplasm. Moreover, cells that bind a biopolymer but cleave away the fluorescently tagged portion of the polysaccharide may not be labeled despite a high level of activity. Second, taxonomic biases may also be introduced by taxon-specific differences in cell lysis efficiency, SSU rRNA gene primer mismatches during SAG PCR, or interference of fluorescent substrates with downstream molecular analyses. For example, we had low success rate recovering DNA from cells labeled with fluorescent xylan (Figure 2). This may be caused by multiple factors, such as xylan inhibition of cell lysis and DNA amplification, or by some of the sorted fluorescent particles being cellulosome-like enzyme complexes [39] or other non-living particles with associated enzymes. Some support for the latter possibility is provided by the notably lower light side scatter (a proxy for particle size) among xylan-labeled particles, as compared to laminarin-labeled particles (Figure 2A). Despite these limitations, which may be addressed by future method improvements, FS-SAGA offers a powerful and cost-effective tool to rapidly identify and recover discrete genomes of active players in biopolymer degradation, without the need for cultivation.

### Conclusions

We demonstrate the use of FS-SAGA to recover genomes of active laminarin and xylan degraders in coastal and freshwater bacterioplankton, opening new opportunities for basic microbial ecology research and for bioprospecting. Our results indicate unexpected significance in polysaccharide hydrolysis of a few relatively rare, yet widely distributed, planktonic *Verrucomicrobia* phylotypes. The employed method could be readily applied to recover genomes of microorganisms involved in the degradation of diverse polysaccharides in a wide range of environments, utilizing well-established protocols for polysaccharide fluorescent labeling [2] and high-throughput single cell genomics [16]–[18]. The spectrum of target substrates may be expanded to other chemical classes, after the development of suitable fluorescent labeling techniques.

## Materials and Methods

### Optimization of cell probing conditions with fluorescently labeled polysaccharides

A surface water sample was collected from Damariscotta Lake (44°10′38″N 69°29′12″W) in Maine, USA, on July 23, 2008 and analyzed within two hours of storage at in situ temperature in the dark. The sample was pre-screened through a 70 µm mesh-size cell strainer (BD), divided into 2 mL aliquots, amended with either 4 µM or 40 µM fluorescein-labeled laminarin (final concentration) and incubated at in situ temperature in the dark. The laminarin was synthesized and labeled with fluoresceinamine as described in detail elsewhere, with about 1 in 148 monomers receiving fluorescent tags [11], [40], [41]. A subsample of the field sample was brought to boil in a microwave oven, cooled down to room temperature, and then aliquoted and amended with fluorescein-labeled laminarin as above, to serve as a killed, negative control. After 5, 12, 20, 60 and 120 minutes of incubation, each treatment was analyzed for the abundance of green-fluorescent particles in the prokaryote size range, using light side scatter as a proxy for particle size. Approximately 10^5^ mL^−1^ of 2.15 µm fluorescent SkyBlue microspheres (Spherotech, Inc., Libertyville, IL) were added to each treatment to serve as internal standards, and their abundance was determined by epifluorescence microscopy. Putative fluorescent microbial cells and fluorescent microspheres were counted in each treatment using a MoFlo™ (Beckman Coulter) flow cytometer. The gate for putative fluorescent cells was delineated in the light side scatter interval that is typical for prokaryotes and in the green fluorescence interval above the background fluorescence. The abundance of putative fluorescently labeled cells per mL sample was estimated as the ratio of gated cell-like particles versus the microsheres, multiplied by the abundance of microspheres and corrected for dilution.

### Sample collection and cell labeling for the main experiment

Surface water samples were collected from the Gulf of Maine (43°50′40″N 69°38′27″W) and the freshwater, mesotrophic Damariscotta Lake (44°10′38″N 69°29′12″W) in Maine, USA, on July 19, 2009 and analyzed within two hours of storage at *in situ* temperature in the dark. Water samples were pre-screened through a 70 µm mesh-size cell strainer (BD) and the bacterioplankton cells were labeled, in parallel, using the following fluorescent probes, for subsequent single cell sorting:

1. Fluoresceinamine-labeled polysaccharides laminarin and xylan (4 µM final concentration, 20–60 min incubation), which were obtained as described above. Only particles with low light side scatter, likely corresponding to individual prokaryote cells, were sorted.
2. SYTO-9 DNA stain (Invitrogen; 5 µM final concentration; 10–120 min incubation) to label all bacterioplankton cells [15]. The high and low nucleic acid content cells of prokaryotes (HNA and LNA) were sorted and processed separately.
3. The 5-cyano-2,3-ditolyltetrazolium chloride (CTC; Sigma; 5 mM final concentration; 60 min incubation) for detection of prokaryotes with active electron transport system (ETS), indicative of cell's viability [21].
4. The carboxyfluorescein diacetate (CFDA; Invitrogen; 10 uM final concentration; 20–60 min incubation) for detection of prokaryotes with intracellular esterase activity, as another proxy of cell's viability [23].

No specific permits were required for the described field studies.

### Single cell sorting, whole genome amplification and PCR

Microbial cells were sorted with a MoFlo™ (Beckman Coulter) flow cytometer equipped with a CyClone™ robotic arm for droplet deposition into 384-well plates. The cytometer was triggered on side scatter. The “single 1 drop" mode was used for maximal sort purity, which ensures the absence of non-target particles within the target cell drop and the adjacent drops. Under these sorting conditions, sorted drops contain a few 10's of pL of sample surrounding the target cell [42], so non-target DNA is very low or absent. The accuracy of 10 µm fluorescent bead deposition into the 384-well plates was verified by microscopically examining the presence of beads in the plate wells. Of the 2–3 plates examined each sort day, <2% wells were found to not contain a bead and only <0.5% wells were found to contain more than one bead, indicating very high purity of single cells. In addition, we verified the lack of DNA contamination in the sheath fluid and in sheath fluid lines by performing real-time multiple displacement amplification with the processed sheath fluid as the template.

Bacterial cells were deposited into 384-well plates containing 0.6 µL per well of TE buffer. Plates were stored at −80°C until further processing. Of the 384 wells, 315 were dedicated for single cells, 66 were used as negative controls (no droplet deposition) and 3 received 10 cells each (positive controls). The cells were lysed and their DNA was denatured using cold KOH [14]. Genomic DNA from the lysed cells was amplified using multiple displacement amplification (MDA) [14], [43] in 10 µL final volume. The MDA reactions contained 2 U/uL Repliphi polymerase (Epicentre), 1× reaction buffer (Epicentre), 0.4 mM each dNTP (Epicentre), 2 mM DTT (Epicentre), 50 mM phosphorylated random hexamers (IDT) and 1 µM SYTO-9 (Invitrogen) (all final concentration). The MDA reactions were run at 30°C for 12–16 h, and then inactivated by 15 min incubation at 65°C. The amplified genomic DNA was stored at −80°C until further processing. We refer to the MDA products originating from individual cells as single amplified genomes (SAGs). To obtain sufficient quantity of genomic DNA for shotgun sequencing of selected SAGs, the original MDA products were re-amplified using similar MDA conditions as above: eight replicate 125 µL reactions were performed and then pooled together, resulting in ∼100 µg of genomic dsDNA for each SAG.

The instruments and the reagents were decontaminated for DNA prior to sorting and MDA setup, as previously described [15], [44]. Cell sorting and MDA setup were performed in a HEPA-filtered environment. As a quality control, the kinetics of each MDA reaction was monitored by measuring the SYTO-9 fluorescence using FLUOstar Omega (BMG). The critical point (Cp) was determined for each MDA reaction as the time required to produce half of the maximal fluorescence. The Cp is inversely correlated to the amount of DNA template [45]. The Cp values were significantly lower in 1-cell wells compared to 0-cell wells (p<0.05; Wilcoxon Two Sample Test) in each microplate.

The MDA products were diluted 50-fold in sterile TE buffer. Then 0.5 µL aliquots of the dilute MDA products served as templates in 5 µL real-time PCR screens targeting bacterial SSU rRNA genes using primers 27F′ and 907R [46], [47]. Forward (5′–GTAAAACGACGGCCAGT-3′) or reverse (5′–CAGGAAACAGCTATGACC–3′) M13 sequencing primer was appended to the 5′ end of each PCR primer to aid direct sequencing of the PCR products. All PCRs were performed using LightCycler 480 SYBR Green I Master mix (Roche) in a LightCycler® 480 II real time thermal cycler (Roche). The real-time PCR kinetics and the amplicon melting curves served as proxies detecting successful target gene amplification. New, 20 µL PCR reactions were set up for the PCR-positive SAGs and the amplicons were sequenced from both ends using M13 targets and Sanger technology by Beckman Coulter Genomics. Single cell sorting, whole genome amplification and real-time PCR screens were performed at the Bigelow Laboratory Single Cell Genomics Center (www.bigelow.org/scgc). Our previous studies and other recent publications using our single cell sequencing technique demonstrate the reliability of our methodology with insignificant levels of DNA contamination in individual cell MDA products [10], [15]–[18], [44], [48]–[51].

### 16S rRNA phylogenetic analysis

The SAG 16S rRNA gene sequences were aligned using the SILVA aligner [52]. Phylogenetic analysis based on maximum likelihood (1000 bootstrap replications) was performed with RAxML version 7.0.3 [53] implemented in ARB package [54], using the reference ARB database 102 containing 460,783 high quality 16S rRNA sequences. The core tree was calculated with the closest reference sequences and then partial sequences from SAGs (742–833 nucleotide positions) were added using the ARB parsimony tool. Those 16S rRNA gene sequences from SAGs that displayed ≥99% similarity were grouped into the same phylotype. Quantitative β-diversity analysis was performed to compare the diversity found in the SAG libraries by using the weighted UniFrac model [55]. For that purpose, a neighbor-joining tree (Jukes-Cantor substitution model), including the 16S rRNA gene sequences from SAGs served as the input data for Fast UniFrac analysis. The archaeon *Halobacterium salinarum* (AB074299) served as an outgroup. Genbank accession numbers of the 16S rRNA gene sequences from SAGs are JF488098–JF488633.

### Whole genome sequencing

Whole genome sequencing was accomplished using a hybrid approach, combining Illumina short read data with PacBio long read data. One microgram aliquots of amplified single cell genomic DNA were prepared following the Illumina TruSeq DNA Sample Preparation Guide for the GAIIx system (Illumina, Revision A, Nov 2010). The completed libraries were validated using the Qubit (Invitrogen Corporation, Carlsbad, CA) for quantitation. Samples ranged from 37 ng/ul to 57 ng/ul. The Agilent Bioanalyzer (Agilent Technolgies, Santa Clara, CA) was used to determine the size of the PCR enriched fragments for all samples. The size range for the samples was from 320 to 540 base pairs. The libraries were normalized to 10 nM, denatured and diluted to 8 pM in preparation for cluster generation on the Illumina Cluster Station using the Paired End Cluster Generation Kit Version 4. During cluster generation, the SAG libraries were multiplexed onto five lanes of the flowcell, three libraries per lane. The flowcell was run on the Illumina GA11x using the TruSeq Paired End Sequencing By Synthesis Kit Version 5–GA with a multiplexed recipe for a 110+7+110 cycle run.

For the PacBio RS data, three microgram aliqots of amplified single cell genomic DNA were acoustically sheared in a Covaris E210 (Covaris©) to a target fragment size of 2 kb using the shearing conditions provided in the Pacific Biosciences Sample Preparation and Sequencing Guide (Pacific Biosciences, 2010–2011). The protocol for preparing a 2 kb library was subsequently followed, using 1 µg of purified, sheared DNA as starting material. Template concentration was calculated using the Qubit fluorometer and the average size was determined by BioAnalyzer trace analysis and served as input to the Annealing & Binding Calculator v.1.2.1 (Pacific Biosciences, March 2011) to prepare SMRTbell-template annealing and polymerase-template binding reactions, as well as the final dilution of the polymerase-bound template complex for sample plate loading and spike-in of control DNA. Due to the variability of sequence data per SMRT cell, we sequenced 6–20 SMRT cells per sample to achieve estimated genome coverage of at least 10×. All cells were sequenced with sequencing movie lengths of 40 minutes. The PacBio reads were filtered to a minimum read length of 100 bp and a minimum read quality score of 0.85.

Assemblies were conducted using the Los Alamos National Laboratory assembly pipeline. Briefly, the Velvet assembler [56] is used for Illumina data using a range of Kmers and coverage cutoffs and the resulting contigs are merged together into a final assembly using in house Perl scripts. This assembly was combined with PacBio data using the PacBio AHA (A Hybrid Assembler) software to incorporate long reads and join contigs. The obtained contigs were subject to another round of assembly, using Sequencher software version 4.10.1 (Gene Codes). Ambiguities were trimmed off the ends and contigs overlapping by at least 100 bp and 98% sequence identity were merged into larger contigs. The resulting draft assemblies were used for subsequent analysis.

To verify the absence of contaminating sequences in the assemblies, tetramer frequencies were extracted from all scaffolds and the Principal Component Analysis (PCA) was then used to extract the most important components of this high dimensional feature matrix [16], [48]. Scaffolds representing extremes on the first eight PCs were manually examined for their closest tblastx hits against NCBI nt database, which did not yield any close hits to non-*Verrucomicrobia* genomes, thus providing no evidence of contamination in the assemblies.

Partial genome assemblies of the five sequenced *Verrucomicrobia* SAGs were submitted to Genbank under accession numbers CAGK00000000, CAGL00000000, CAGM00000000, CAGN00000000, GACO00000000. The raw shotgun sequences of the five *Verrucomicrobia* SAGs were deposited in the NCBI short read archive under accession numbers ERP001168 for Illumina reads and ERP001168 for PacBio reads.

### Genome annotation and comparative genomics

Prediction of open reading frames was performed with GenMark [57]. Glycoside hydrolase genes were automatically annotated using the CAZymes Analysis Toolkit applying the association rule learning algorithm [58]. The resulting annotation was carefully revised by using conserved domain BLAST [59], BLASTp against non redundant proteins and the resources of SWISS-MODEL [60], PROSITE [61], and CAZy databases [26]. Bioinformatic resources of the Integrated Microbial Genomes (IMG) system were used to estimate the frequency of glycoside hydrolase genes (E.C. 3.2.1.x; see CAZy database) in the publicly available prokaryote genomes in the IMG database (http://img.jgi.doe.gov/cgi-bin/m/main.cgi) as of February 2012. Frequency was calculated for each bacterial genome by dividing the total number of genes annotated as glycoside hydrolases by the total number of annotated genes for that particular genome. Then, the average enrichment of glycoside hydrolases for each bacterial phylum was estimated. Peptidase genes were annotated using MEROPS peptidases database [28].

Estimates of complete genome sizes were obtained using conserved single copy gene (CSCG) analysis [48]. To identify relevant CSCGs, 6 genomes from the *Verrucomicrobia* phylum, currently available at the Joint Genome Institute Integrated Microbial Genomes site [62], were included in the analysis: *Akkermansia muciniphila* ATCC BAA-835, *Coraliomargarita akajimensis* DSM 45221, *Methylacidiphilum infernorum* V4, *Opitutus terrae* PB90-1, *Verrucomicrobiales* sp. DG1235, and *Verrucomicrobium spinosum* DSM 4136. Of the COG function distributions listed in these genomes, 273 CSCGs were found to be shared by all 6 finished or draft sequences. Of the 273 identified CSCGs, 87 (31.9%), 151 (55.3% ), 168 (61.5%), 199 (72.9%), and 239 (87.6% ) were present in the SAGs using rps-blast against the COG database, which correlated with assembly size (1.0 Mb, 2.1 Mb, 2.6 Mb, 3.3 Mb, and 4.9 Mb respectively). The expected genome sizes for each SAG was estimated using the function G_s_ = A_s_/R_CSCG_, where G_S_ is the expected complete genome size; A_S_ is the size of the SAG assemblies; R_CSCG_ is the recovery of CSCGs based on COG analysis. Thus, the expected genome sizes of these five SAGs are estimated to be approximately 3.2 Mb, 3.8 Mb, 4.4 Mb, 4.7 Mb, and 5.7 Mb.

Pairwise genome comparisons of average nucleotide identity (ANI) [63] and tetranucleotide signature [64] were performed with Jspecies [65], after genome alignment with MUMmer [66] and BLAST [67].

## Supporting Information

Figure S1 **Taxonomic composition of freshwater single amplified genomes (SAGs).** Bacterioplankton were probed with the nucleic acid stain SYTO-9, representing a random subset of the total microbial assemblage, and with fluoresceinamine-labeled polysaccharides laminarin and xylan.
(TIF)

Figure S2 **Phylogenetic composition of polysaccharide-positive** ***Gammaproteobacteria, Acidobacteria, Bacteroidetes, Planctomycetes, OP10*** **, and** ***OP11*** **.** (A) Maximum likelihood phylogenetic analysis of the SSU rRNA gene sequences. Bootstrap (1000 replicates) values ≥50 are displayed. Each phylotype, indicated in red (coastal) or blue (freshwater) is formed by SAGs with ≥99% SSU rRNA gene sequence similarity. (B) Phylotype relative abundances in SAG libraries generated using various fluorescent probes. (nd) = not detected in a SAG library.
(TIF)

Figure S3 **Principal coordinate analysis (PCoA) of weighted pairwise UniFrac distances between SSU rRNA gene sequences from the various coastal bacterial fractions.** Included are high nucleic acid content (HNA), low nucleic acid content (LNA), ETS-active and esterase-active cells. A Neighbor-Joining tree employing Jukes-Cantor substitution model served as the input data.
(TIF)

Figure S4 **Frequency of glycoside hydrolase genes in** ***Verrucomicrobia*** **genomes.** (A) Relationship between the abundance of glycoside hydrolase genes and genome size in the publicly available *Verrucomicrobia* genomes. (B) Relationship between the abundance of glycoside hydrolase genes and the genome size in the five sequenced *Verrucomicrobia* SAGs. (C) The number of genes encoding carbohydrate-active enzymes, glycoside transferases and sulfatases in the five sequenced SAGs of the phylotype AAA168-F10.
(TIF)

Figure S5 **Comparative genome analysis of the five sequenced single amplified genomes (SAGs) of the** ***Verrucomicrobia*** **phylotype AAA168-F10.** Plotted are values of the average nucleotide identity (ANI) and the tetranucleotide frequency signature for each of the pairwise genome comparisons.
(TIF)

Figure S6 **Evidence for the xylanase gene in the single amplified genome AAA168-F10.** (A) Active site, including the catalytic residues responsible for xylan hydrolysis, derived from Conserved Domain Protein, SWISS-MODEL, and PROSITE databases. (B) Neighbor-joining phylogenetic tree of amino acid sequences, applying Kimura evolutionary model and indicating bootstrap values above 50.
(TIF)

Figure S7 **Signal peptide prediction for laminarinase protein sequence.** Prediction of signal peptide was performed with SignalP 3.0 Server. Cleavage site is indicated at the N-terminus of the protein sequence, which is used to direct the protein through the cellular membrane.
(TIF)

Figure S8 Peptidase genes encoded by the single amplified genome AAA168-F10. A total of 67 peptidase families were found. Peptidases acting on polypeptides (e.g., family M1) and oligopeptides (e.g., S9), carboxy/aminopeptidases (e.g., M14/M42), dipeptidyl-peptidases (e.g., S15) and endopeptidases (e.g., S01B) are encoded on the AAA168-F10 genome. Annotation was performed using the MEROPS peptidase database.
(TIF)

Table S1 **Summary of single amplified genomes (SAGs) from which the 16S rRNA gene was recovered.**
(DOC)

Table S2 **Abundance of polysaccharide-positive** ***Verrucomicrobia*** **phylotypes among ETS- and esterase-positive, coastal SAGs.**
(DOC)

Table S3 **Assembly statistics.**
(DOC)

Table S4 **Glycoside hydrolase enzymes encoded by SAG AAA168-F10.**
(DOC)
