# Supplementary figures and images for: Capturing Single Cell Genomes of Active Polysaccharide Degraders: An Unexpected Contribution of Verrucomicrobia
Source: PLoS One. 2012 Apr 20;7(4):e35314. doi: 10.1371/journal.pone.0035314 (PMC3335022; doi:10.1371/journal.pone.0035314)

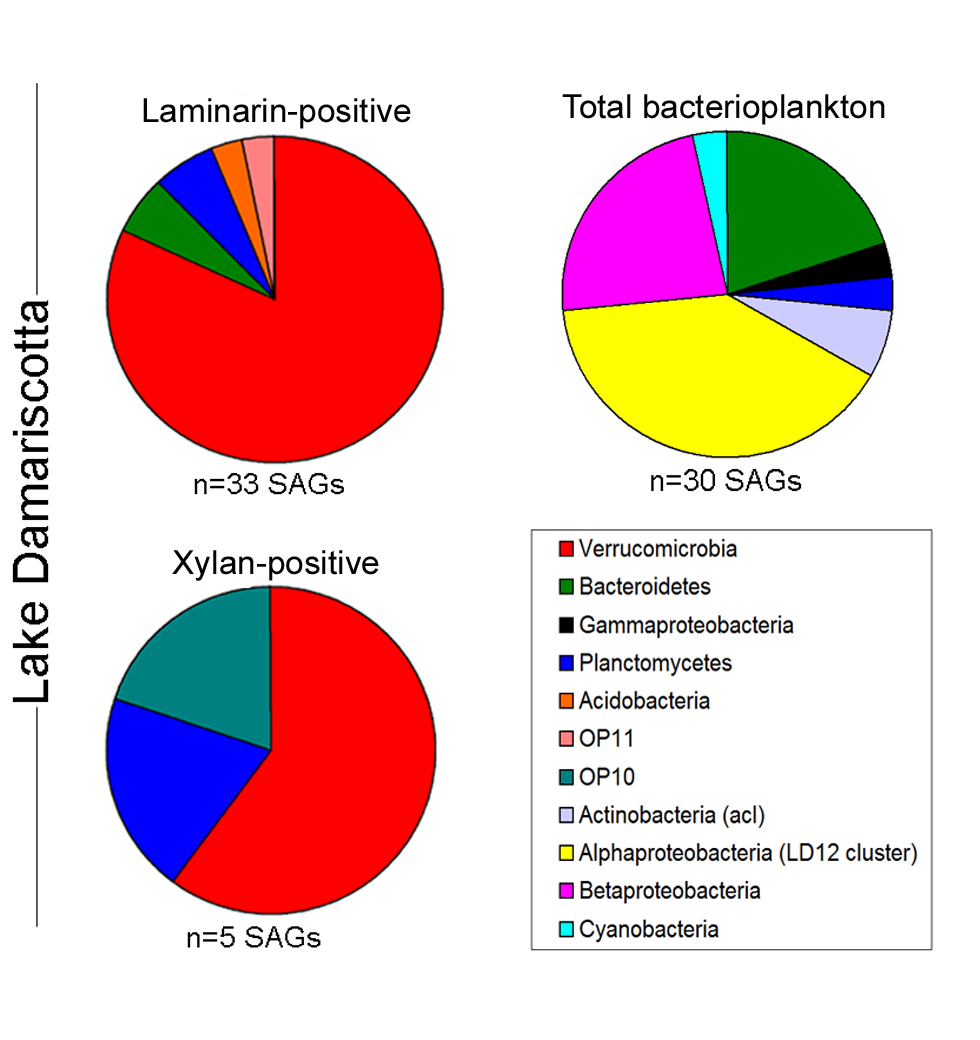

Supplement: Figure S1 — Taxonomic composition of freshwater single amplified genomes (SAGs). Bacterioplankton were probed with the nucleic acid stain SYTO-9, representing a random subset of the total microbial assemblage, and with fluoresceinamine-labeled polysaccharides laminarin and xylan. (TIF) [file pone.0035314.s001.tif]

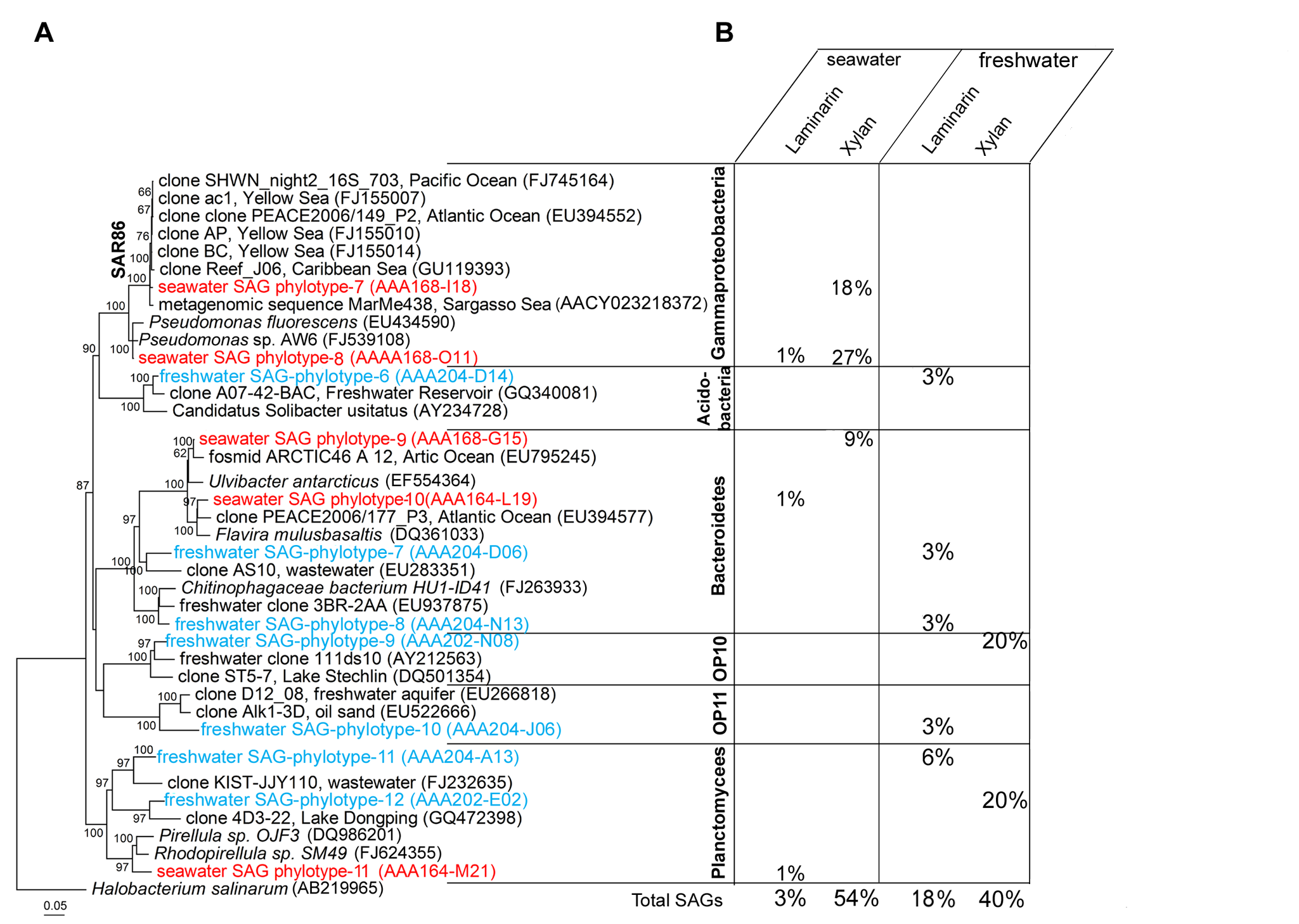

Supplement: Figure S2 — Phylogenetic composition of polysaccharide-positive Gammaproteobacteria, Acidobacteria, Bacteroidetes, Planctomycetes, OP10 , and OP11 . (A) Maximum likelihood phylogenetic analysis of the SSU rRNA gene sequences. Bootstrap (1000 replicates) values ≥50 are displayed. Each phylotype, indicated in red (coastal) or blue (freshwater) is formed by SAGs with ≥99% SSU rRNA gene sequence similarity. (B) Phylotype relative abundances in SAG libraries generated using various fluorescent probes. (nd) = not detected in a SAG library. (TIF) [file pone.0035314.s002.tif]

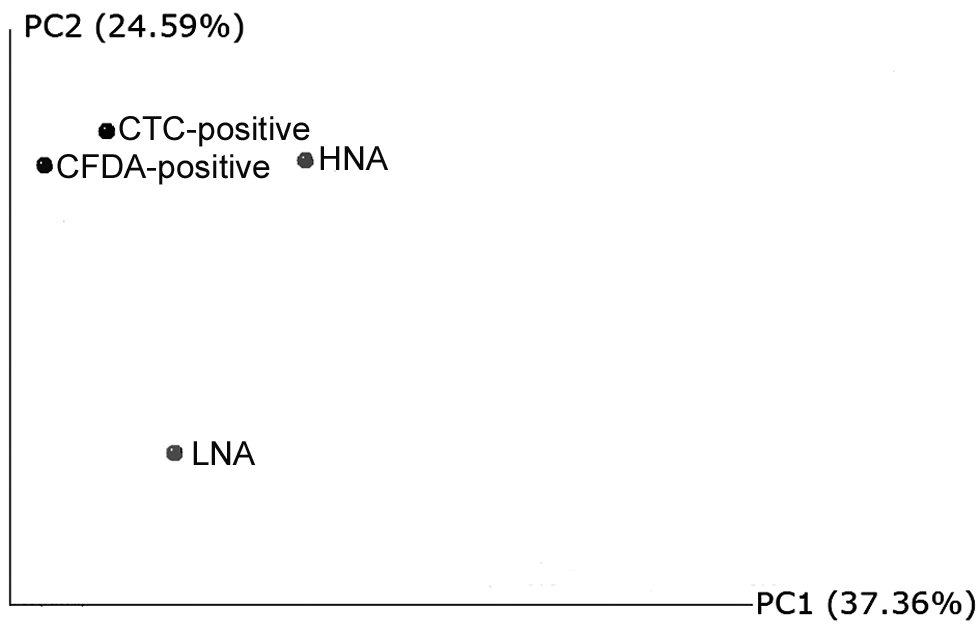

Supplement: Figure S3 — Principal coordinate analysis (PCoA) of weighted pairwise UniFrac distances between SSU rRNA gene sequences from the various coastal bacterial fractions. Included are high nucleic acid content (HNA), low nucleic acid content (LNA), ETS-active and esterase-active cells. A Neighbor-Joining tree employing Jukes-Cantor substitution model served as the input data. (TIF) [file pone.0035314.s003.tif]

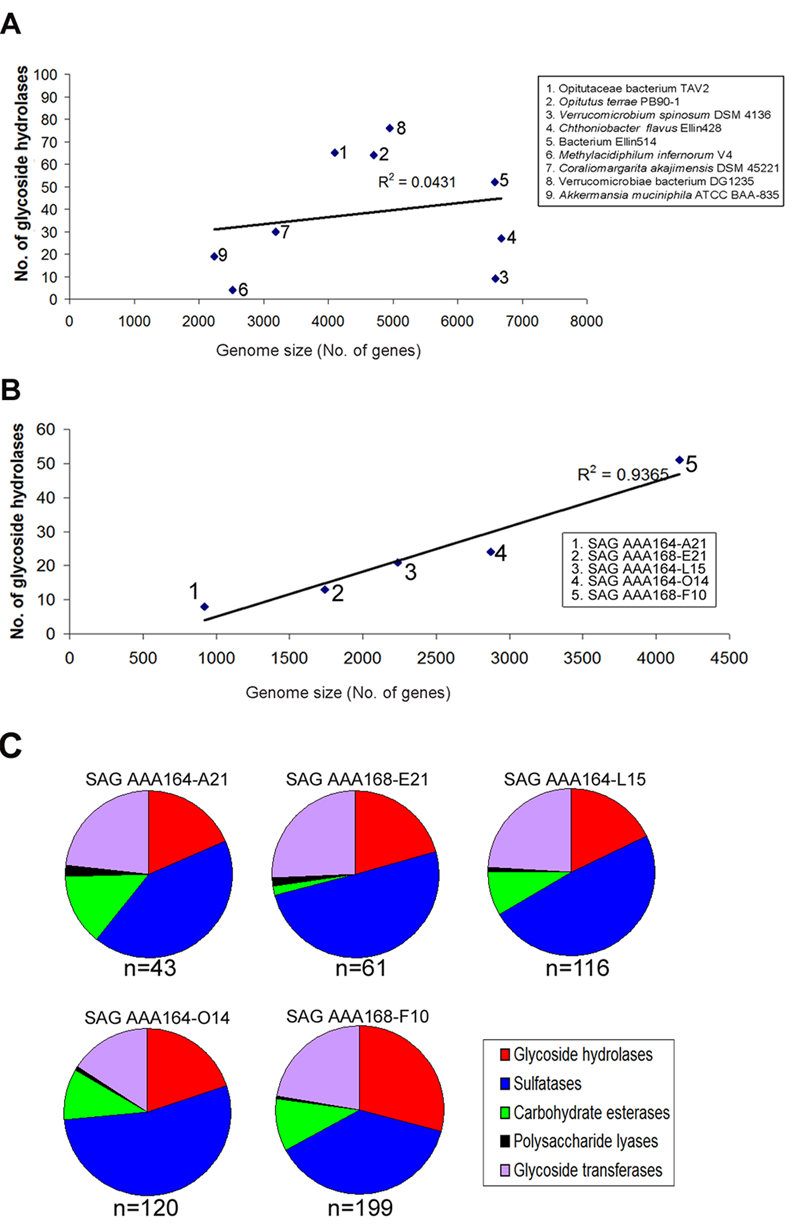

Supplement: Figure S4 — Frequency of glycoside hydrolase genes in Verrucomicrobia genomes. (A) Relationship between the abundance of glycoside hydrolase genes and genome size in the publicly available Verrucomicrobia genomes. (B) Relationship between the abundance of glycoside hydrolase genes and the genome size in the five sequenced Verrucomicrobia SAGs. (C) The number of genes encoding carbohydrate-active enzymes, glycoside transferases and sulfatases in the five sequenced SAGs of the phylotype AAA168-F10. (TIF) [file pone.0035314.s004.tif]

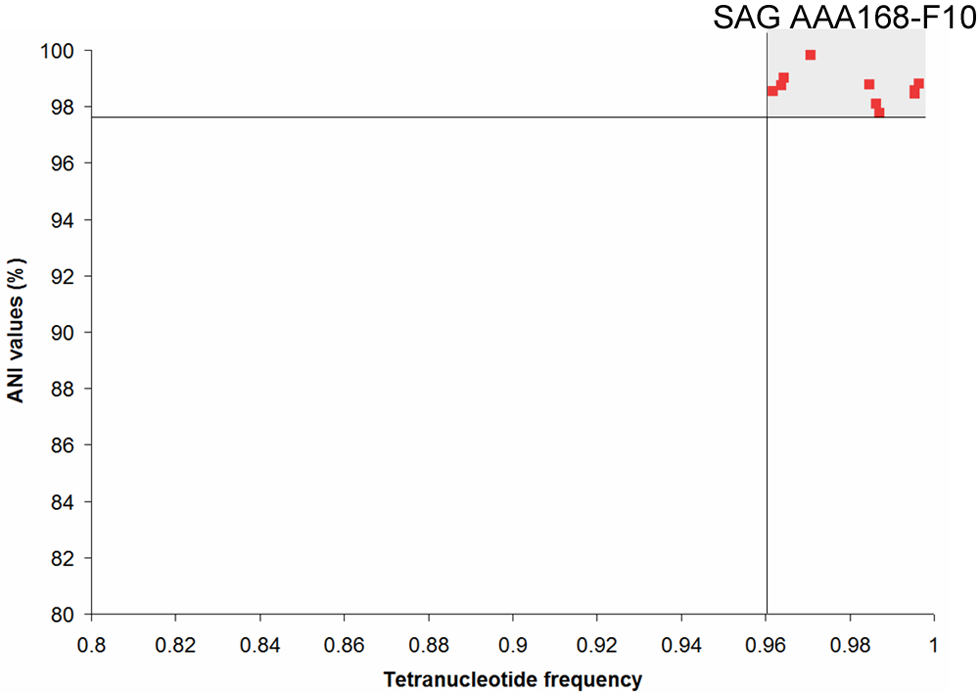

Supplement: Figure S5 — Comparative genome analysis of the five sequenced single amplified genomes (SAGs) of the Verrucomicrobia phylotype AAA168-F10. Plotted are values of the average nucleotide identity (ANI) and the tetranucleotide frequency signature for each of the pairwise genome comparisons. (TIF) [file pone.0035314.s005.tif]

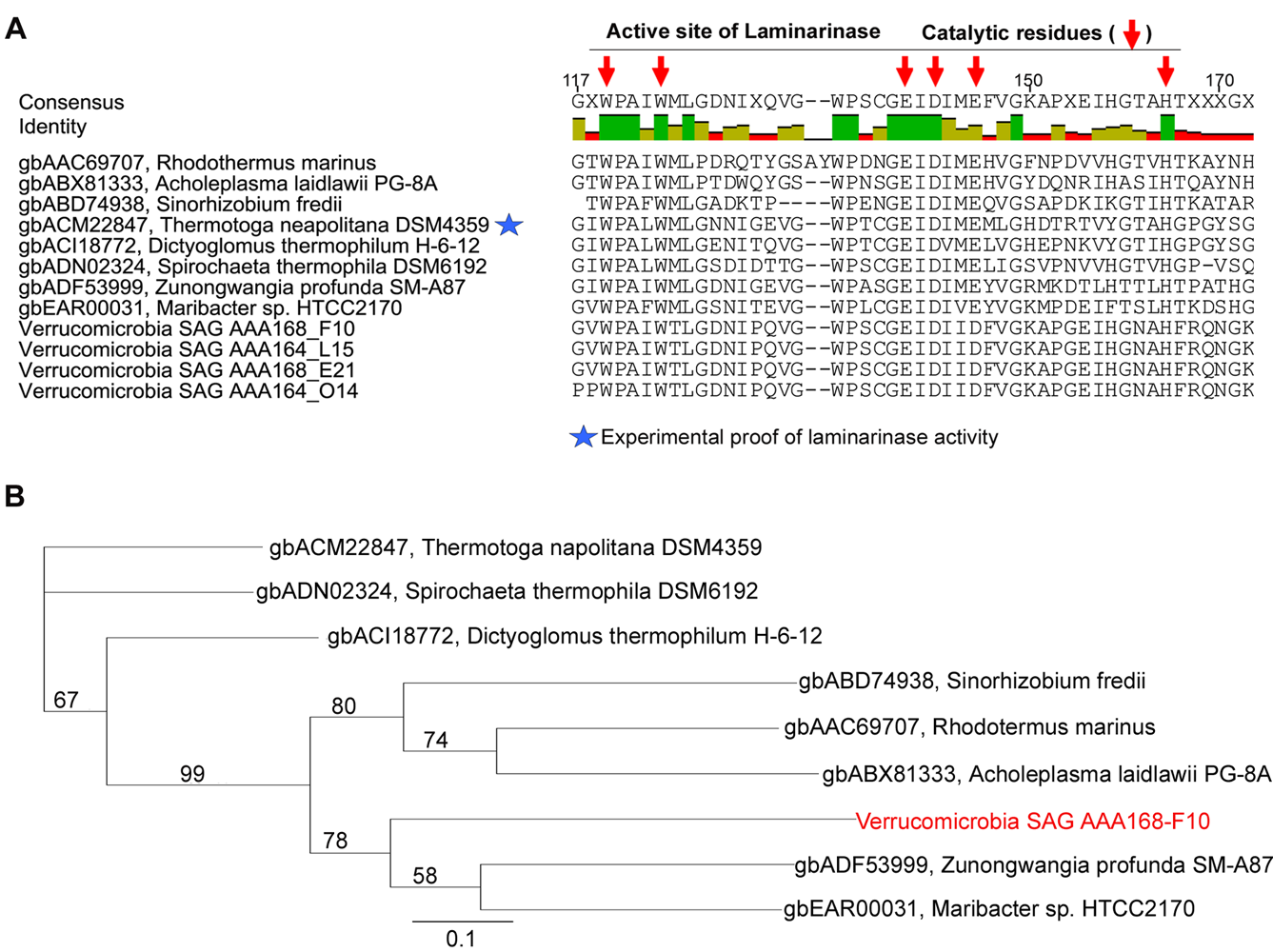

Supplement: Figure S6 — Evidence for the xylanase gene in the single amplified genome AAA168-F10. (A) Active site, including the catalytic residues responsible for xylan hydrolysis, derived from Conserved Domain Protein, SWISS-MODEL, and PROSITE databases. (B) Neighbor-joining phylogenetic tree of amino acid sequences, applying Kimura evolutionary model and indicating bootstrap values above 50. (TIF) [file pone.0035314.s006.tif]

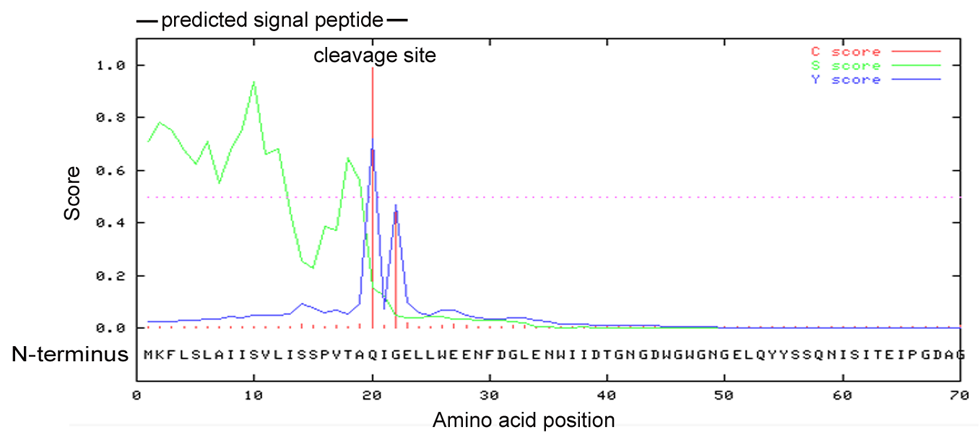

Supplement: Figure S7 — Signal peptide prediction for laminarinase protein sequence. Prediction of signal peptide was performed with SignalP 3.0 Server. Cleavage site is indicated at the N-terminus of the protein sequence, which is used to direct the protein through the cellular membrane. (TIF) [file pone.0035314.s007.tif]

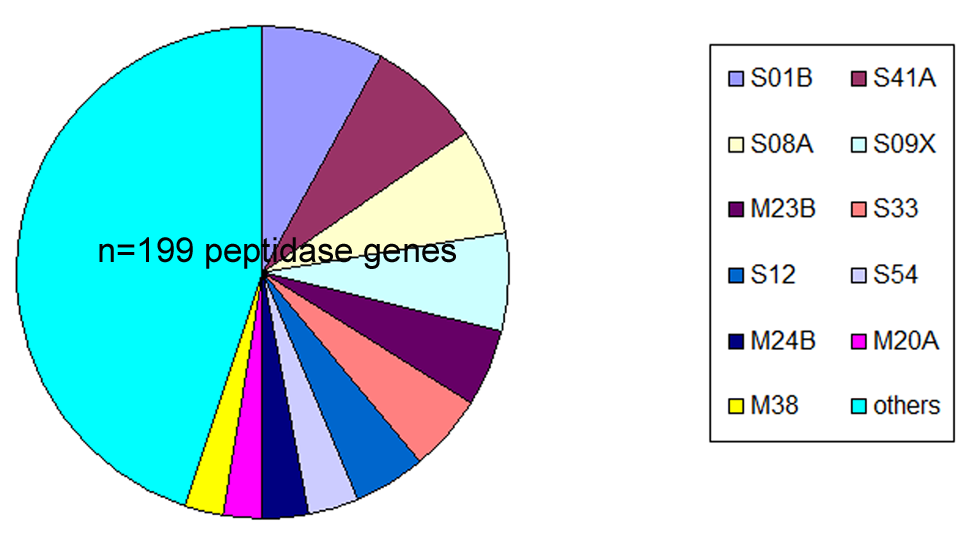

Supplement: Figure S8 — Peptidase genes encoded by the single amplified genome AAA168-F10. A total of 67 peptidase families were found. Peptidases acting on polypeptides (e.g., family M1) and oligopeptides (e.g., S9), carboxy/aminopeptidases (e.g., M14/M42), dipeptidyl-peptidases (e.g., S15) and endopeptidases (e.g., S01B) are encoded on the AAA168-F10 genome. Annotation was performed using the MEROPS peptidase database. (TIF) [file pone.0035314.s008.tif]
