# Supplementary material for: Capturing Single Cell Genomes of Active Polysaccharide Degraders: An Unexpected Contribution of Verrucomicrobia
Source: PLoS One. 2012 Apr 20;7(4):e35314. doi: 10.1371/journal.pone.0035314 (PMC3335022; doi:10.1371/journal.pone.0035314)
Supplement: Table S1 — Summary of single amplified genomes (SAGs) from which the 16S rRNA gene was recovered. (DOC) [file pone.0035314.s009.doc]

| Environment | SAG libraries | SAG label | SAGs^a^ |
| --- | --- | --- | --- |
| Gulf of Maine | Laminarin-positive | (AAA164) | 121 (38%) |
|  | Xylan-positive | (AAA168) | 11 (4%) |
|  | HNA (Total bacterioplankton) | (AAA160) | 106 (34%) |
|  | LNA (Total bacterioplankton) | (AAA163) | 50 (16%) |
|  | ETS-positive | (AAA158) | 47 (15%) |
|  | Esterase-positive | (AAA076) | 68 (21%) |
|  | Total coastal |  | 414 (22%) |
| Lake Damariscotta | Laminarin-positive | (AAA204) | 33 (10%) |
|  | Xylan-positive | (AAA202) | 5 (2%) |
|  | HNA (Total bacterioplankton) | (AAA206) | 16 (5%) |
|  | LNA (Total bacterioplankton) | (AAA208) | 14 (4%) |
|  | Total lake |  | 68 (4%) |
| ^a^SAGs yielding SSU rRNA gene sequence, fraction of all single cell MDA products in parentheses. | | | |
