# Supplementary material for: Capturing Single Cell Genomes of Active Polysaccharide Degraders: An Unexpected Contribution of Verrucomicrobia
Source: PLoS One. 2012 Apr 20;7(4):e35314. doi: 10.1371/journal.pone.0035314 (PMC3335022; doi:10.1371/journal.pone.0035314)
Supplement: Table S2 — Abundance of polysaccharide-positive Verrucomicrobia phylotypes among ETS- and esterase-positive, coastal SAGs. (DOC) [file pone.0035314.s010.doc]

|  | | |
| --- | --- | --- |
| SAG name | ETS-positive SAG | esterase-positive SAGs |
| SAG phylotype-1 (AAA168-F10) | 6% | nd^a^ |
| SAG phylotype-2 (AAA164-L23) | nd | nd |
| SAG phylotype-3 (AAA164-J09) | nd | nd |
| SAG phylotype-4 (AAA164-L05) | 2% | nd |
| SAG phylotype-5 (AAA164-E20) | nd | nd |
| Total | 8% | nd |
| ^a^Not detected in the analyzed SAG library | | |
