# Supplementary material for: Capturing Single Cell Genomes of Active Polysaccharide Degraders: An Unexpected Contribution of Verrucomicrobia
Source: PLoS One. 2012 Apr 20;7(4):e35314. doi: 10.1371/journal.pone.0035314 (PMC3335022; doi:10.1371/journal.pone.0035314)
Supplement: Table S3 — Assembly statistics. (DOC) [file pone.0035314.s011.doc]

|  | | | | | |
| --- | --- | --- | --- | --- | --- |
|  | AAA164-A21 | AAA168-E21 | AAA164-L15 | AAA164-O14 | AAA168-F10 |
| Assembly size (bp) | 1,011,436 | 2,060,559 | 2,641,408 | 3,346,464 | 4,850,044 |
| Estimated genome recovery (%) | 31.9 | 55.3 | 61.5 | 72.9 | 87.6 |
| Number of contigs | 754 | 1084 | 710 | 1141 | 1397 |
| Largest contig (bp) | 34216 | 31946 | 116022 | 57159 | 92178 |
| GC content (%) | 48 | 49 | 49 | 49 | 49 |
| Illumina sequencing effort (Mbp) | 3276 | 2582 | 2187 | 2705 | 2554 |
| PacBio sequencing effort (Mbp) | 328 | 476 | 429 | 170 | 430 |
| Total predicted genes | 920 | 1741 | 2237 | 2870 | 4157 |
