# Supplementary material for: Capturing Single Cell Genomes of Active Polysaccharide Degraders: An Unexpected Contribution of Verrucomicrobia
Source: PLoS One. 2012 Apr 20;7(4):e35314. doi: 10.1371/journal.pone.0035314 (PMC3335022; doi:10.1371/journal.pone.0035314)
Supplement: Table S4 — Glycoside hydrolase enzymes encoded by SAG AAA168-F10. (DOC) [file pone.0035314.s012.doc]

|  | | | | |
| --- | --- | --- | --- | --- |
| CAZy family | No. of genes | Enzyme | Putative substrates | Best BLASTp hit  (bit score/ evalue) |
| GH2 | 2 | (2) β-glucuronidase (EC [3.2.1.31](http://www.enzyme-database.org/query.php?ec=3.2.1.31)) | β-D-[glucuronic acid](http://en.wikipedia.org/wiki/Glucuronic_acid) ([glycosaminoglycans](http://en.wikipedia.org/wiki/Glycosaminoglycan)/ mucopolysaccharides) | *Bacteroides* sp. [ZP_06201167](http://www.ncbi.nlm.nih.gov/entrez/query.fcgi?cmd=Retrieve&db=Protein&list_uids=270294966&dopt=GenPept)  ([114](../../../../C:%5CUsers%5Cmanuel%5CDesktop%5CResults_RSE10_GH_BLASTp_html.html#319955075) 4e-024) |
| GH3 | 5 | (2) β-N-acetylhexosaminidase (EC [3.2.1.52](http://www.enzyme-database.org/query.php?ec=3.2.1.52))  (2) β-glucosidase (EC [3.2.1.21](http://www.enzyme-database.org/query.php?ec=3.2.1.21))  (1) glucan 1,4-β-glucosidase (EC [3.2.1.74](http://www.enzyme-database.org/query.php?ec=3.2.1.74)) | Hexosamines  beta-D-glucosides (β-D-galactosides, α-L-arabinosides, β-D-xylosides, β-D-fucosides)  1,4-β-D-glucans and related oligosaccharides | *Chlorobium phaeobacteroides* [YP_912990](http://www.ncbi.nlm.nih.gov/entrez/query.fcgi?cmd=Retrieve&db=Protein&list_uids=119358346&dopt=GenPept)  ([294](../../../../C:%5CUsers%5Cmanuel%5CDesktop%5CResults_RSE10_GH_BLASTp_html.html#119358346) / 1e-077)  *Zunongwangia profunda* [YP_003585551](http://www.ncbi.nlm.nih.gov/entrez/query.fcgi?cmd=Retrieve&db=Protein&list_uids=295134875&dopt=GenPept)  ([380](../../../../C:%5CUsers%5Cmanuel%5CDesktop%5CResults_RSE10_GH_BLASTp_html.html#295134875) / e-103)  *Maricaulis maris* [P_757950](http://www.ncbi.nlm.nih.gov/entrez/query.fcgi?cmd=Retrieve&db=Protein&list_uids=114571270&dopt=GenPept)  ([936](../../../../C:%5CUsers%5Cmanuel%5CDesktop%5CResults_RSE10_GH_BLASTp_html.html#114571270) / 0.0) |
| GH5 | 3 | Cellulase family A (including endo-1,4-β-xylanase EC [3.2.1.8](http://www.enzyme-database.org/query.php?ec=3.2.1.8)) | Cellulose and hemicellulose | *Polaribacter irgensii* [ZP_01118913](http://www.ncbi.nlm.nih.gov/entrez/query.fcgi?cmd=Retrieve&db=Protein&list_uids=88803387&dopt=GenPept)  ([177](../../../../C:%5CUsers%5Cmanuel%5CDesktop%5CResults_RSE10_GH_BLASTp_html.html#88803387) / 7e-043) |
| GH9 | 3 | cellobiohydrolase (EC [3.2.1.91](http://www.enzyme-database.org/query.php?ec=3.2.1.91)) | cellulose and cellotetraose | uncultured organism [ACY24809](http://www.ncbi.nlm.nih.gov/entrez/query.fcgi?cmd=Retrieve&db=Protein&list_uids=262089714&dopt=GenPept) ([458](../../../../C:%5CUsers%5Cmanuel%5CDesktop%5CResults_RSE10_GH_BLASTp_html.html#262089714) / e-126) |
| GH10 | 3 | endo-1,4-β-xylanase (EC [3.2.1.8](http://www.enzyme-database.org/query.php?ec=3.2.1.8)) | xylan | Candidatus *Solibacter usitatus* [YP_823955](http://www.ncbi.nlm.nih.gov/entrez/query.fcgi?cmd=Retrieve&db=Protein&list_uids=116621799&dopt=GenPept)  ([217](../../../../C:%5CUsers%5Cmanuel%5CDesktop%5CResults_RSE10_GH_BLASTp_html.html#116621799) / 9e-055) |
| GH13 | 8 | (1) cyclomaltodextrinase (EC [3.2.1.54](http://www.enzyme-database.org/query.php?ec=3.2.1.54))  (7) α-amylase (EC [3.2.1.1](http://www.enzyme-database.org/query.php?ec=3.2.1.1)) | cyclomaltodextrin  starch, glycogen and related polysaccharides and oligosaccharides | uncultured soil bacterium [ABA39290](http://www.ncbi.nlm.nih.gov/entrez/query.fcgi?cmd=Retrieve&db=Protein&list_uids=76097012&dopt=GenPept)  ([474](../../../../C:%5CUsers%5Cmanuel%5CDesktop%5CResults_RSE10_GH_BLASTp_html.html#76097012) / e-131)  *Croceibacter atlanticus* [003715784|](http://www.ncbi.nlm.nih.gov/entrez/query.fcgi?cmd=Retrieve&db=Protein&list_uids=298207605&dopt=GenPept)  ([595](../../../../C:%5CUsers%5Cmanuel%5CDesktop%5CResults_RSE10_GH_BLASTp_html.html#298207605) / e-168) |
| GH16 | 1 | glucan endo-1,3-β-glucosidase or laminarinase (EC [3.2.1.39](http://www.enzyme-database.org/query.php?ec=3.2.1.39)) | laminarin | *Zunongwangia profunda* SM-A87 [YP_003584827.1](http://www.ncbi.nlm.nih.gov/entrez/query.fcgi?cmd=Retrieve&db=Protein&list_uids=295134151&dopt=GenPept) ([147](../../../../C:%5CUsers%5Cmanuel%5CDesktop%5CResults_RSE10_GH_BLASTp_html.html#295134151) / 5e-034) |
| GH17 | 1 | glucan endo-1,3-β-glucosidase (EC [3.2.1.](http://www.enzyme-database.org/query.php?ec=3.2.1.39)x) | (1→3)-β-D-glucans Lichenin | Verrucomicrobiae bacterium [ZP_05057481](http://www.ncbi.nlm.nih.gov/entrez/query.fcgi?cmd=Retrieve&db=Protein&list_uids=254444005&dopt=GenPept)  ([165](../../../../C:%5CUsers%5Cmanuel%5CDesktop%5CResults_RSE10_GH_BLASTp_html.html#254444005) / 2e-039) |
| GH26 | 1 | β-mannanase (EC [3.2.1.78](http://www.enzyme-database.org/query.php?ec=3.2.1.78)) | mannans, galactomannans and glucomannans | *Rhodothermus marinus* [YP_003289757](http://www.ncbi.nlm.nih.gov/entrez/query.fcgi?cmd=Retrieve&db=Protein&list_uids=268316038&dopt=GenPept)  ([260](../../../../C:%5CUsers%5Cmanuel%5CDesktop%5CResults_RSE10_GH_BLASTp_html.html#268316038) / 6e-067) |
| GH31 | 1 | α-glucosidase (EC [3.2.1.20](http://www.enzyme-database.org/query.php?ec=3.2.1.20)) | Polysaccharides with (1→4)-α-glucosidic linkages | *Lacinutrix* sp. AEH02520  (836 / 0.0) |
| GH43 | 3 | xylosidase/arabinosidase (EC [3.2.1.37](http://www.enzyme-database.org/query.php?ec=3.2.1.37), EC [3.2.1.55](http://www.enzyme-database.org/query.php?ec=3.2.1.55)) | xylan , arabinans | *Bacteroides plebeius* [ZP_03207375](http://www.ncbi.nlm.nih.gov/entrez/query.fcgi?cmd=Retrieve&db=Protein&list_uids=198274843&dopt=GenPept)  ([674](../../../../C:%5CUsers%5Cmanuel%5CDesktop%5CResults_RSE10_GH_BLASTp_html.html#198274843) / 0.0) |
| GH77 | 1 | Amylomaltase or 4-alpha-glucanotransferase  (EC [2.4.1.25](http://www.enzyme-database.org/query.php?ec=2.4.1.25)) | maltose | *Geobacter sulfurreducens* [NP_952235](http://www.ncbi.nlm.nih.gov/entrez/query.fcgi?cmd=Retrieve&db=Protein&list_uids=39996284&dopt=GenPept)  ([182](../../../../C:%5CUsers%5Cmanuel%5CDesktop%5CResults_RSE10_GH_BLASTp_html.html#39996284) / 1e-044) |
| GH78 | 2 | α-L-rhamnosidase (EC [3.2.1.40](http://www.enzyme-database.org/query.php?ec=3.2.1.40)) | α-L-rhamnose | *Rhodopirellula baltica* [EGF24665](http://www.ncbi.nlm.nih.gov/entrez/query.fcgi?cmd=Retrieve&db=Protein&list_uids=327537969&dopt=GenPept)  ([79](../../../../C:%5CUsers%5Cmanuel%5CDesktop%5CResults_RSE10_GH_BLASTp_html.html#327537969) / 3e-013) |
| GH81 | 5 | endo-β-1,3-glucanase (EC [3.2.1.39](http://www.enzyme-database.org/query.php?ec=3.2.1.39)) | laminarin | *Micromonospora aurantiaca* ([YP_003836446](http://www.ncbi.nlm.nih.gov/entrez/query.fcgi?cmd=Retrieve&db=Protein&list_uids=302867809&dopt=GenPept))  ([198](../../../../C:%5CUsers%5Cmanuel%5CDesktop%5CResults_RSE10_GH_BLASTp_html.html#302867809) / 5e-049) |
| GH109 | 19 | α-N-acetylgalactosaminidase (EC [3.2.1.49](http://www.enzyme-database.org/query.php?ec=3.2.1.49)) | N-acetylgalactosamine (glycoproteins from cellular surface and cell wall) | *Akkermansia muciniphila* [YP_001877822](http://www.ncbi.nlm.nih.gov/protein/187735710?report=genbank&log$=prottop&blast_rank=1&RID=ZMAJ5YMS014)  (313 / 3e-83) |
|  |  |  |  |  |
